# Patterns of radiological response to procarbazine and CCNU polychemotherapy in patients with oligodendroglioma

**DOI:** 10.1093/noajnl/vdag184

**Published:** 2026-08-06

**Authors:** Sophie Katzendobler, Luis Kuschel, Nicola Zieger, Frederic Thiele, Michael Schmutzer-Sondergeld, Dragan Jankovic, Roman Stürzl, Patrick N Harter, Robert Stahl, Darius Kalasauskas, Asgeir S Jakola, Nathalie L Albert, Emilie Le Rhun, Michael Weller, Florian Ringel, Jonathan Weller

**Affiliations:** Department of Neurosurgery, LMU University Hospital, LMU Munich, Munich, Germany; Department of Neurosurgery, LMU University Hospital, LMU Munich, Munich, Germany; Department of Neurosurgery, LMU University Hospital, LMU Munich, Munich, Germany; Department of Neurosurgery, LMU University Hospital, LMU Munich, Munich, Germany; Department of Neurosurgery, LMU University Hospital, LMU Munich, Munich, Germany; Department of Neurosurgery, LMU University Hospital, LMU Munich, Munich, Germany; Department of Nuclear Medicine, LMU University Hospital, LMU Munich, Munich, Germany; Center for Neuropathology and Prion Research, LMU Munich, Munich, Germany; Institute for Diagnostic and Interventional Neuroradiology, University Hospital, LMU Munich, Munich, Germany; Department of Neurosurgery, LMU University Hospital, LMU Munich, Munich, Germany; Department of Neurosurgery, Sahlgrenska University Hospital, Gothenburg, Sweden; Institute of Clinical Sciences, Sahlgrenska Academy, Gothenburg, Sweden; Department of Nuclear Medicine, LMU University Hospital, LMU Munich, Munich, Germany; Department of Medical Oncology and Hematology, University Hospital and University of Zurich, Zurich, Switzerland; Department of Neurology, University Hospital and University of Zurich, Zurich, Switzerland; Department of Neurosurgery, LMU University Hospital, LMU Munich, Munich, Germany; Department of Neurosurgery, LMU University Hospital, LMU Munich, Munich, Germany

**Keywords:** grade, IDH, low, neoadjuvant, volumetry

## Abstract

**Purpose:**

To characterize the radiological response to procarbazine and CCNU (chloroethyl-cyclohexyl-nitrosourea) (PC) polychemotherapy in patients with oligodendroglioma.

**Methods:**

In this retrospective single-center cohort study, patients with Central Nervous System World Health Organization (CNS WHO) grade 2 or 3 oligodendroglioma treated with PC between 2003 and 2019 were included. Tumor characteristics were assessed on magnetic resonance imaging (MRI) and [^18^F]Fluoroethyltyrosine positron emission tomography ([^18^F]FET PET) before and after completion of PC. The T1/T2 ratio was assessed to characterize diffuse versus sharply delineated MRI phenotype. Progression-free survival (PFS) and overall survival (OS) were analyzed.

**Results:**

Forty-six patients with a median follow-up of 118 months were identified. Median absolute T2 tumor volume was 52 cm³ (range 10-285) prior to PC and 29 cm³ (range 3-286) after. Median tumor volume decrease was −30% (range −96% to +113%). In treatment-naïve patients, median tumor volume decrease was −41% (range −92% to +14%) versus −7% (range −96% to +113%) in pretreated patients (*P* < .01). Stratification according to CNS WHO grade (*P* = .89) or contrast enhancement (*P* = .07) did not yield significant differences. The T1/T2 ratio was lower after PC (0.69 vs 0.50, *P* = .04), and higher T1/T2 ratios were associated with shorter PFS (*P* = .04) in multivariate analysis. On [^18^F]FET PET, there was a trend towards lower maximum tumor-to-brain ratios (TBRmax) after PC (*P* = .07).

**Conclusion:**

PC polychemotherapy induces substantial tumor volume reductions in subsets of patients with oligodendroglioma, particularly in treatment-naïve patients. The observations that circumscript oligodendrogliomas may be associated with earlier progression and that PC treatment may induce a shift toward a more diffuse phenotype require prospective validation.

Key Points

- PC polychemotherapy induces a substantial tumor volume decrease in a subset of patients with oligodendroglioma, especially in treatment-naïve patients.
- PC polychemotherapy might induce a phenotype switch towards a slightly more diffuse appearance on MRI as assessed by the T1/T2 ratio.
- A sharply delineated phenotype on MRI is associated with shorter progression-free survival.

Importance of the Study

Despite the established role of PC polychemotherapy in patients with oligodendroglioma, the magnitude and determinants of chemotherapy-induced tumor volume changes remain poorly characterized. In this study, we performed quantitative magnetic resonance imaging (MRI)-based volumetric analyses before and after PC polychemotherapy and integrated these measurements with assessment of tumor imaging phenotype using the T1/T2 ratio. This allowed us to characterize patterns of radiological response and identify clinical factors associated with treatment effect and progression.

Standard treatment for patients with newly diagnosed oligodendroglioma comprises tumor resection followed by observation or radiotherapy followed by procarbazine, CCNU (lomustine) and vincristine (PCV) polychemotherapy.^1^ Patients with oligodendroglioma are typically young to middle-aged adults who show few or no symptoms in the early stages of the disease.^2-4^ Survival can extend to decades even with expectative management strategies initially, and preservation of neurological function and independency in activities of daily living should be considered carefully in the care of these patients.^2^^,^^5^^,^^6^ Age below 40, absence of fixed neurological deficits and low tumor burden have traditionally been considered as criteria justifying a wait-and-scan strategy,^7^ but these data predate the recognition of isocitrate dehydrogenase (IDH) mutations as a major prognostic factor. With the emergence of new promising therapeutic agents, notably mutant IDH inhibitors such as vorasidenib, alternative strategies become available for subsets of patients not considered candidates for immediate chemoradiotherapy.^8^

While the risks of postoperative deficits and radiotherapy-induced toxicity might depend on the tumor volume and involvement of eloquent areas, those of systemic therapies do not. *Neoadjuvant* strategies have been implemented a core principle for the treatment of numerous neoplasms such as breast cancer, rectal adenocarcinoma, soft-tissue sarcoma and non-small cell lung cancer.^9-12^ Similar controlled studies for patients with gliomas have not yet been conducted, but chemotherapy-induced tumor volume decreases have been reported in small case series of patients with IDH-mutant gliomas.^13-16^ The extent of these changes, including potential tumor growth despite chemotherapy, and its association with prior tumor-specific therapies and potential risk factors for shorter survival such as contrast enhancement on MRI, tumor size or Central Nervous System World Health Organization (CNS WHO) grade remain incompletely defined.

A modified regimen of procarbazine and CCNU (PC) without vincristine is administered at our institution—in lieu of PCV—in order to avoid the risk of vincristine-associated peripheral neuropathy.^17^ This present study explored PC-induced tumor volume changes as the main outcome parameter in subgroups of patients with oligodendrogliomas, stratified by patient- and tumor-related risk factors. We sought to evaluate the potential role of PC polychemotherapy as a neoadjuvant therapy for selected patients based on magnetic resonance imaging (MRI) and, if available, [^18^F]Fluoroethyltyrosine positron emission tomography ([^18^F]FET PET). Beyond the assessment of tumor volumes, we investigated the MRI phenotype before and after PC polychemotherapy by means of the T1/T2 ratio, a recently published parameter used to quantitively describe a diffuse versus sharp appearance on MRI.^18^ The T1/T2 ratio represents the quotient of the absolute T1-hypointense tumor volume divided by the T2-hyperintense tumor volume. Sharply delineated gliomas appear as hypointense lesions on T1-weighted sequences and as hyperintense lesions of similar size on T2-weighted sequences.^19^^,^^20^ In such cases, the T1/T2 ratio is close to 1. In contrast, gliomas that appear diffuse on MRI typically show much smaller volumes on T1-weighted sequences, resulting in a T1/T2 ratio that approaches 0. A cutoff value of 0.33 has been reported to indicate inferior resectability and overall survival (OS) in patients with astrocytoma.^18^^,^^21^

## Methods

### Patient Evaluation and Histopathology

The study was conducted with the approval of the institutional ethics committee of the Ludwig Maximilian University Hospital Munich (project number 20-513). The local database of the Center for Neuropathology was searched for patients diagnosed with glioma with IDH mutations and 1p/19q codeletion between 2003 and 2019 and reclassified according to the CNS WHO Classification 2021 (Supplementary Figure S1).^3^ Molecular status of 1p/19q was assessed by means of microsatellite marker analysis.^22^  *IDH1* and *IDH2* mutations were confirmed by immunohistochemical staining and pyrosequencing in all patients. Inclusion criteria comprised history of PC polychemotherapy and available MRI data at baseline and within three months after completion of the last PC cycle. PC polychemotherapy was given for 6 cycles if tolerated, following dosing regimens from prospective clinical trials.^23^^,^^24^ Due to the risk of peripheral polyneuropathy, vincristine was not routinely administered for the treatment of oligodendroglioma at our institution but left to physician and patient preference. Patients treated with PC or PCV were pooled and termed “PC,” as only 4 patients received vincristine. The baseline scan was the last scan before initiation of PC. Patients who did not have a tumor resection underwent a stereotactic, frame-based biopsy for tissue sampling.^25^^,^^26^ In patients having undergone tumor resection, the baseline MRI scan had to show measurable disease according to RANO 2.0 definition, ie ≥ 1 cm non-enhancing or enhancing tumor in all three orthogonal directions.^27^ Medical records were investigated and patient-related data assessed. Clinical status was quantified by Karnofsky performance status as part of the clinical routine.

Progression-free survival (PFS) was defined as the interval from initiation of PC polychemotherapy to disease progression, as determined by clinical and radiological evaluation and corresponding multidisciplinary tumor board recommendations. Therapeutic interventions comprised tumor resection, radiotherapy, or temozolomide therapy. Complications from PC polychemotherapy were evaluated utilizing the Common Terminology Criteria for Adverse Events (CTCAE) version 5.^28^ OS was defined as the time from diagnosis by histological sampling until date of death. The time from initiation of PC polychemotherapy to death was termed post-PC survival.

### Volumetric Measurements and Imaging Parameters

All patients received MRI scans on 1.5 or 3.0 Tesla MRI scanners prior to tissue acquisition and after termination of PC polychemotherapy. T1-weighted sequences with and without contrast agent (gadopentetate dimeglumin) as well as T2-sequences had to be available in all patients. Contrast enhancement was evaluated in accordance with the Visually AcceSAble Rembrandt Images approach (VASARI) (REMBRANDT—The Cancer Imaging Archive [TCIA]) as not present, mild/minimal, or marked/avid. Tumor segmentation was done in a semi-automated fashion on T2-weighted MRI sequences as well as T1-weighted sequences without contrast agent by expert raters utilizing Elements Smartbrush software (Brainlab AG, Munich, GER). The T1/T2 ratio was calculated before and after PC polychemotherapy. Response to and progression after PC polychemotherapy on MRI was assessed retrospectively according to RANO 2.0 (Response Assessment in Neuro-Oncology) criteria for enhancing and non-enhancing gliomas.^27^ For non-enhancing lesions, responses included complete response (CR), partial response (PR), minor response (MR), stable disease (SD), and progressive disease (PD), and for gliomas with enhancing and non-enhancing components, all the above except for MR.^27^

If available before and after PC polychemotherapy, sequential [^18^F]FET PET images were investigated. To this end, a local Hermes workstation was used (Hermes Medical Solutions, Stockholm, Sweden). Mean and maximum standardized uptake values (SUVmean/SUVmax) were measured. The PET-positive tumor volume was segmented on the basis of a 1.6-fold SUVmean background threshold as recommended by PET RANO 1.0.^29^ The mean and maximum tumor-to-brain ratio (TBRmean/TBRmax) were calculated by dividing the tumor SUVs by background SUVmean. To determine response, PET RANO 1.0 categories were applied.^29^

### Statistics

The primary objective of this study was to assess radiological response to PC polychemotherapy as measured by changes in tumor volume. Secondary endpoints included PFS, OS, and RANO-based response. The T1/T2 ratio was analyzed as an imaging biomarker and exploratory variable. Statistical analyses were computed using GraphPad Prism 10.5.0 (GraphPad Software Inc., Boston, USA). Comparison of categorical variables was performed using the Chi-square test or Fisher’s exact test, as appropriate. When global tests revealed significant differences across more than two groups, pairwise post-hoc comparisons were conducted using Fisher’s exact test with Bonferroni correction for multiple testing. Continuous variables were compared using the unpaired t-test for normally distributed data and the Mann-Whitney U test for non-normally distributed data. Normal distribution was assessed using D’Agostino’s K-squared test. Correlations between non-parametric datasets were assessed using Spearman’s correlation coefficient, whereas Pearson’s correlation coefficient was calculated for normally distributed data. Cox proportional hazards regression was used to determine associations between patient-related parameters and PFS after PC as well as OS in univariate and multivariate analyses. Hazard ratios (HR) and 95% confidence intervals (95% CI) were calculated. Variables with a significant *P*-value in univariate analyses were entered into multivariate analyses. A two-sided *P*-value ≤ .05 was considered statistically significant.

## Results

### Patient Characteristics

We identified 46 patients diagnosed between 2003 and 2019 who met inclusion criteria. Median age at diagnosis was 39 (range 19-75) (Table 1 and Supplementary Figure S1). Stereotactic biopsy without tumor resection prior to PC polychemotherapy was performed in 42 patients (91%). A tumor resection was performed in 4 patients (9%). A CNS WHO grade 2 was diagnosed in 39 patients (85%) and a grade 3 in 7 patients (15%). Contrast enhancement on MRI was seen in 31 patients (67%). The contrast enhancement was mild in 17 patients (37%) and avid in 14 (30%) (Table 1). Seventeen patients (37%) received PC polychemotherapy without prior radiotherapy or temozolomide chemotherapy. Nineteen patients (41%) had already received temozolomide therapy, and 22 patients (48%) had been treated with radiotherapy. Radiotherapy and temozolomide therapy had been given to 12 patients (26%) prior to PC. PC was administered in 4 patients (9%) and PC without vincristine in 42 patients (91%). The intended number of cycles was 6. The median number of cycles completed was 6 (range 2-6). The median time from completion of the last PC cycle to MRI was 1 month (range 0-3). PC was halted for toxicity in 12 patients (26%) and for PD in 6 patients (13%). In three patients, a tumor resection was performed due to PD under PC. In 2 patients, radiotherapy was performed, and in 1 patient, temozolomide was initiated. There was no grade 4 or 5 adverse event according to CTCAE v5.0 in the entire cohort. In 18 patients (39%), no adverse event was reported. Grade 1 adverse events were reported in 16 patients (35%), grade 2 in 4 patients (9%), and grade 3 in 8 patients (17%). The most frequent adverse events were leukopenia (*n* = 9; 20%), thrombocytopenia (*n* = 7, 15%), and fatigue (*n* = 7; 15%).

**Table 1.** Baseline characteristics

Parameter	All patients (*n* = 46)
Age (years)	
Median	39
Range	19-75
Sex, n (%)	
Female	20 (43)
Male	26 (57)
KPS prior to PC	
Median	90
Range	70-100
Type of prior therapies beyond surgery	
None	17 (37)
Only TMZ	7 (15)
Only RT	10 (22)
RT/TMZ combined	0
RT and TMZ sequentially (separate lines)	12 (26)
Prior tumor-specific therapies, *n* (%)	
0	13 (28)
1	14 (30)
2	10 (22)
≥3	9 (20)
Main location, *n* (%)	
Frontal	28 (61)
Insular	5 (11)
Temporal	8 (17)
Parietal	4 (9)
Occipital	1 (2)
Midline	0 (0)
CNS WHO grade, *n* (%)	
2	39 (85)
3	7 (15)
Contrast enhancement, *n* (%)	
No	15 (33)
Mild/minimal	17 (37)
Marked/avid	14 (30)
PC cycles, *n*	
Median	6
Range	2-6
RANO 2.0 response, *n* (%)	
Complete response	0 (0)
Partial response	8 (17)
Minor response	3 (7)
Stable disease	29 (63)
Progressive disease	6 (13)
Initial T2 volume (cm^3^)	
Median	52
Range	10-285
T2 volume after PC polychemotherapy (cm^3^)	
Median	29
Range	3-286
Initial T1/T2 ratio	
Median	0.69
Range	0.03-1.0
**T1/T2 ratio after PC polychemotherapy**	
Median	0.5
Range	0.01-1.0

Prior tumor-specific therapies comprise tumor resection, radiotherapy and temozolomide chemotherapy. PC, procarbazine and CCNU (lomustine); KPS, Karnofsky performance status; TMZ, temozolomide; RANO, Response Assessment in Neuro-Oncology; RT, radiotherapy; CNS, central nervous system; WHO, World Health Organization.

### Response Patterns to PC Polychemotherapy

**Figure 1.**
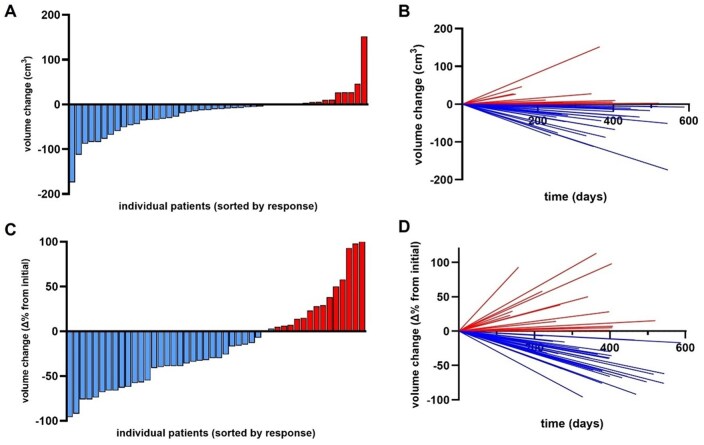
Waterfall (A, C) and spider (B, D) plots for absolute (A, B) and relative (C, D) tumor volume changes associated with PC polychemotherapy in patients with oligodendroglioma. The response at the end of PC polychemotherapy is shown. PC, procarbazine and CCNU (lomustine); Δ, delta/difference; cm^3^, cubic centimeter.

Best responses by RANO 2.0 were partial (*n* = 8) or MR (*n* = 3) in 11 patients (24%), SD in 29 patients (63%), and PD in 6 patients (13%) (Table 1). There was no CR. Pairwise Fisher tests with Bonferroni correction confirmed that patients with PD had received significantly more prior therapies than patients with PR/MR (*P* = .006) or SD (*P* = .02). PR or MR were more frequent in female patients (64%) than in male patients (36%), although this difference was not significant (*P* = .24) (Supplementary Table S1). Median tumor volume before initiation of PC polychemotherapy was 52 cm^3^ (range 10-285). Median tumor volume after PC polychemotherapy, ie the tumor volume measured on the MRI scan that was obtained at the end of the last PC cycle, was 29 cm^3^ (range 3-286) (Table 1 and Figure 1). Relative median tumor volume decrease was −30% (range: −96% to +113%). Thirteen patients (28%) showed a volume decrease of more than −50%. In radiotherapy- and temozolomide-naïve patients (*n* = 17; 37%), median tumor volume decrease was −41% (range: −92% to +14%) and more extensive than in patients with prior radiotherapy or temozolomide therapy (*n* = 29; 63%) who had a median tumor volume decrease of −7% (range: −96% to +113%) (*P* < .01). In patients with non-contrast-enhancing oligodendrogliomas, median tumor volume decrease was −35% (range: −76% to +15%). This decrease was not significantly different from contrast-enhancing tumors (median: −21%; range: −96% to +113%) (*P* = .07). Patients with CNS WHO grade 2 oligodendrogliomas did not show a greater extent of volume decrease than patients with CNS WHO grade 3 oligodendrogliomas (median: −30% versus −34%, *P* = .89). In a subgroup analysis of patients pretreated with temozolomide only or radiotherapy only, no significant differences were seen (prior temozolomide only versus radiotherapy only, median: −17% versus +4%, *P* = .69). In patients treated with radiotherapy and chemotherapy, the median volume change was 0.0% (range −68% to +113%).

**Figure 2.**
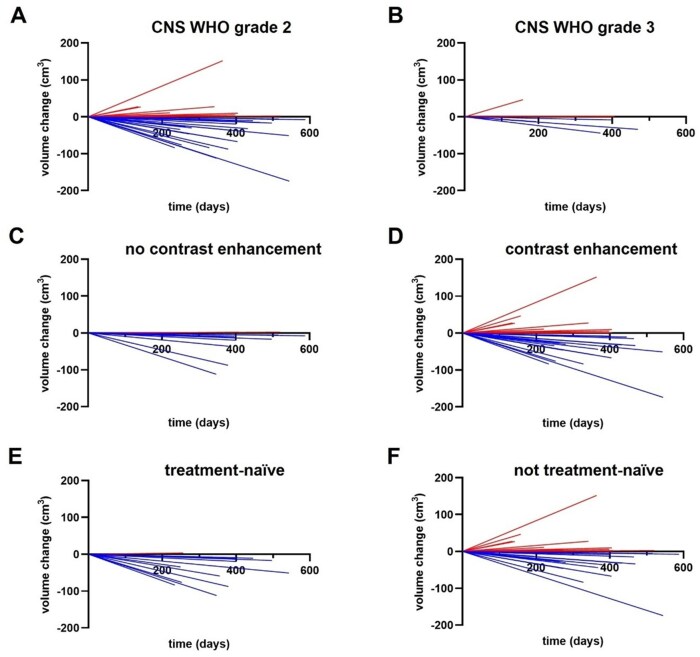
Spider plot showing absolute tumor volume changes after completion or discontinuation of PC polychemotherapy. Subgroups of patients with oligodendrogliomas are shown, stratified by CNS WHO tumor grade (A, B); contrast enhancement on magnetic resonance imaging (C, D) and treatment status (E, F). PC, procarbazine and CCNU (lomustine); CNS, central nervous system; WHO, World Health Organization; Δ, delta/difference; cm^3^, cubic centimeter.

When looking at absolute volume changes, there was a better response in radio- and temozolomide-naïve patients with a median decrease of −34.4 cm^3^ (range: −112.0 cm^3^ to +3.6 cm^3^) versus a median decrease of −4.7 cm^3^ (range: −174.3 cm^3^ to +151.8 cm^3^) in patients having received those therapies at any time before PC (*P* = .01) (Figure 2). There was no significant difference when comparing absolute values of non-contrast-enhancing to contrast-enhancing oligodendrogliomas (median: −8.4 cm^3^ versus −11.9 cm^3^; *P* = .75) or CNS WHO grade 2 to CNS WHO grade 3 oligodendrogliomas (median: −10.8 cm^3^ versus −8.4 cm^3^; *P* = .59) (Figure 2). Non-contrast-enhancing, radio- and temozolomide-naïve oligodendrogliomas (*n* = 8, 17%) showed a median tumor volume decrease of −49% (range: −76% to +7%).

The median T1/T2 ratio before therapy decreased from 0.69 (range 0.03-1.0) to 0.5 (range 0.01-1.0) after PC therapy was (*P* = .04).^18^

There was a trend toward lower TBRmax values with a median of 2.98 before PC polychemotherapy and 2.31 thereafter (*P* = .07). TBRmean showed a non-significant median decrease from 1.95 before to 1.89 after PC (*P* = .48). Median PET volume was 10.5 cm^3^ before PC and 4.2 cm^3^ after (*P* = .85). According to PET RANO 1.0, 6 patients (43%) showed a PR, while 5 (36%) and 3 patients (21%) showed a stable or PD, respectively.

A lower number of previous lines of treatment moderately correlated with a larger extent of volume decrease (r = 0.5, *P* < .01). The pre-PC tumor volume did not correlate with relative tumor volume changes (r=−0.15; *P* = .33) but with absolute tumor volume changes (r=−0.55, *P* < .01). The number of PC cycles completed did not correlate with relative or absolute tumor volume change (r=−0.28; *P* = .06 and r=−0.14; *P* = .34, respectively). Tumor size did not correlate with the T1/T2 ratio (r = 0.07; *P* = .68). Neither did absolute (r = 0.14; *P* = .4) or relative (r = 0.02; *P* = .92) tumor volume changes.

### Outcome Associations of Response Patterns

Median PFS was 41 months, and progression was documented in 30 patients (65%). At database closure, 15 patients (33%) had died. Median overall follow-up for alive patients was 118 months. On univariate analysis, larger absolute tumor volumes after PC polychemotherapy (HR 1.01; 95% CI, 1.0-1.01; *P* = .03) and a higher T1/T2 ratio (HR 4.44; 95% CI, 1.37-15.84; *P* = .01) were associated with shorter PFS. Minimal or absence of CE on MRI (HR 0.59; 95% CI, 0.35-0.97; *P* = .04) and a higher number of PC cycles completed (HR 0.71; 95% CI, 0.56-0.92; *P* = .01) were associated with better PFS (Table 2). Only a lower/higher T1/T2 ratio persisted as a prognostic factor on multivariate analysis (per 1.0-unit increase: HR 3.66; 95% CI, 1.09-13.28; *P* = .04) (Table 2). This corresponded to an approximately 14% increase in hazard per 0.1 increase in the T1/T2 ratio. Larger tumor volumes before (HR 1.01; 95% CI, 1.0-1.01; *P* = .04) and after (HR 1.01; 95% CI, 1.0-1.01; *P* = .02) PC polychemotherapy as well as CE on MRI prior to PC polychemotherapy (HR 0.57; 95% CI, 0.25-0.97; *P* = .04) showed an association with OS on univariate analysis, but not on multivariate analysis (Table 3). Lower KPS, larger tumor volumes after PC polychemotherapy, CE and higher number of prior tumor-specific therapies were associated with shorter post-PC survival in univariate analysis. In multivariate analysis, larger absolute T2 volumes after PC were associated with shorter post-PC survival (Supplementary Table S2).

**Table 2.** Uni- and multivariate analyses for progression-free survival

Univariate analysis			
Factor	PFS		
	HR	95% CI	*P*-value
**Age^x^**	1.01	0.98-1.05	*.49*
**KPS^x^**	0.97	0.93-1.03	*.33*
**CNS WHO grade (3 versus 2)**	1.2	0.43-5.1	*.75*
**Contrast enhancement (marked versus minimal versus no)**	0.59	0.35-0.97	.04*
**T2 volume before PC^x^**	1.00	1.0-1.01	*.22*
**T2 volume after PC^x^**	1.01	1.0-1.01	.03*
**Volume change (absolute)^x^**	1.00	1.0-1.01	*.47*
**Volume change (relative)^x^**	1.53	0.7-3.1	*.27*
**T1/T2 ratio before PC^x^**	4.44	1.37-15.84	.01*
**T1/T2 ratio after PC^x^**	1.44	0.47-4.37	*.52*
**PC cycles^(n)^**	0.71	0.56-0.92	.01*
**Prior tumor-specific therapies^x^**	1.25	0.98-1.56	*.08*

Multivariate analysis			
Factor	PFS		
	HR	95% CI	*P*-value
**Contrast enhancement (marked versus minimal versus no)**	0.74	0.41-1.33	*.31*
**T2 volume after PC^x^**	1.0	1.0-1.01	*.4*
**T1/T2 ratio before PC^x^**	3.66	1.09-13.28	.04*
**PC cycles^(n)^**	0.84	0.59-1.23	*.41*

Prior tumor-specific therapies comprise tumor resection, radiotherapy and temozolomide chemotherapy. PFS, progression-free survival; HR, hazard ratio; CI, confidence interval; KPS, Karnofsky performance status; CNS, central nervous system; WHO, World Health Organization; PC, procarbazine and CCNU (lomustine); n, number of cycles. Statistical significance: *P* < .05 (*). Continuously scaled variables are denoted by ^x^. Italic values indicate *P*-values. Bold values indicate statistically significant results (*P* < .05).

**Table 3.** Uni- and multivariate analyses for overall survival

Univariate analyses			
Factor	OS		
	HR	95% CI	*P*-value
**Age^x^**	1.02	0.98-1.07	*.36*
**KPS^x^**	0.97	0.92-1.02	*.21*
**CNS WHO grade (3 versus 2)**	0.29	0.1-2.8	*.32*
**Contrast enhancement (marked versus minimal versus no)**	0.57	0.25-0.97	.04*
**T2 volume before PC^x^**	1.01	1.0-1.01	.04*
**T2 volume after PC^x^**	1.01	1.0-1.01	.02*
**Volume change (absolute)^x^**	1.00	0.99-1.02	*.84*
**Volume change (relative)^x^**	2.07	0.72-5.67	*.17*
**T1/T2 ratio before PC^x^**	0.83	0.17-4.37	*.82*
**T1/T2 ratio after PC^x^**	2.71	0.6-13.89	*.2*
**PC cycles^(n)^**	0.75	0.52-1.1	*.14*
**Prior tumor-specific therapies^x^**	1.32	0.96-1.77	*.08*

Multivariate analyses			
Factor	OS		
	HR	95% CI	*P*-value
**Contrast enhancement (marked versus minimal versus no)**	0.6	0.28-1.23	*.16*
**T2 volume before PC^x^**	1.0	0.99-1.01	*.99*
**T2 volume after PC^x^**	1.01	1.00-1.02	*.22*

Prior tumor-specific therapies comprise tumor resection, radiotherapy and temozolomide chemotherapy. OS, overall survival; HR, hazard ratio; CI, confidence interval; KPS, Karnofsky performance status; CNS, central nervous system; WHO, World Health Organization; PC, procarbazine and CCNU (lomustine); n, number of cycles. Statistical significance: *P* < .05 (*). Continuously scaled variables are denoted by ^**x**^. Italic values indicate *P*-values. Bold values indicate statistically significant results (*P* < .05).

## Discussion

**Figure 3.**
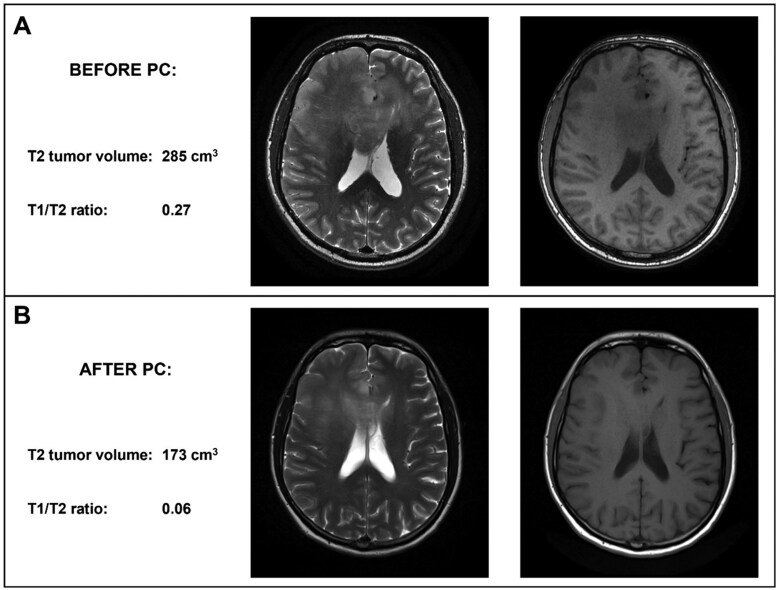
T2- and T1-weighted MRI scans of patients diagnosed with oligodendroglioma prior to PC polychemotherapy (A) and after discontinuation (B). MRI, magnetic resonance imaging; PC, procarbazine and CCNU (lomustine).

PC polychemotherapy is an established treatment option for patients with newly diagnosed and recurrent oligodendroglioma, but its time-dependent effect on tumor size and appearance on MRI remains understudied. We here investigated relative and absolute tumor volumes before and after PC polychemotherapy as well as the imaging phenotype (diffuse versus sharply delineated) by means of the T1/T2 ratio.^18^ Patients were stratified according to potential risk factors to determine subgroup-specific effects. We report a substantial tumor volume decrease in a subset of patients (Figures 1 and 3). Treatment-naïve patients showed a more extensive tumor volume decrease, and the number of prior treatments—ie radio- and temozolomide therapies—negatively correlated with volume decrease. Accordingly, PC polychemotherapy might be especially effective in reducing tumor volume early in the disease course. There was no differential effect of PC polychemotherapy when stratifying for contrast enhancement or CNS WHO grade.

There is only limited data on “neoadjuvant” administration of chemotherapy regimens in patients with oligodendroglioma.^13^^,^^14^ While guidelines allow for the individual consideration of chemotherapy regimens without radiotherapy in younger patients with large tumors, this strategy might also be useful in select patients with smaller tumors and infiltration of eloquent areas.^1^ No treatment-naïve patient showed PD while on PC polychemotherapy (Supplementary Table S1).

The role of resection is pivotal in the treatment of these patients, and supramaximal resection might provide additional survival benefit.^30^^,^^31^ A preoperative tumor volume reduction might enable more extensive resections and potentially counteract invasion of eloquent brain regions.^30^

Our data did not show an association of CNS WHO grade with response to PC, consistent with the notion that grading of oligodendrogliomas remains challenging and that grading introduces an arbitrary cutoff into a continuous disease spectrum. However, regarding CE, a differential effect of PC cannot be excluded based on our data. There was a trend toward more extensive relative tumor volume decrease in patients without contrast enhancement (*P* = .07), and when looking selectively at treatment-naïve patients without contrast enhancement, the tumor volume decrease was especially extensive with a median decrease of −49%. No patient with PD according to RANO 2.0 showed CE before initiation of PC. Further studies with larger cohorts are needed to determine the predictive role of CE for the radiological response to PC. The prognostic importance of absolute tumor volumes in patients with oligodendrogliomas is supported by our study since post-PC tumor volume was associated with post-PC survival in multivariate analysis (Supplementary Table S2).^30^ Absolute tumor volume decrease correlated with the initial tumor size, but relative decrease did not. Accordingly, PC polychemotherapy might be similarly effective in large and in smaller tumors and tumor size was not associated with PC resistance. A higher initial T1/T2 ratio, ie patients with a sharply delineated oligodendroglioma phenotype on MRI, were associated with significantly shorter PFS, even in multivariate analysis. The biological mechanisms underlying both differential appearance on MRI and potential differences in PFS are unknown. The shorter PFS of sharply delineated oligodendrogliomas was not mirrored by OS (Tables 2 and 3). As the median T1/T2 ratio was lower after PC polychemotherapy, there might be a PC-induced shift toward a slightly more diffuse phenotype (Table 1). Studies correlating imaging phenotype with histological features and molecular signatures are urgently needed. The median T1/T2 ratio was 0.5 after therapy. A threshold at 0.33 has been established as a cutoff for limited resectability and shorter OS in patients with astrocytoma.^18^^,^^21^ The clinical implications of the T1/T2 ratio, both regarding resectability and prognostication, has not yet been investigated in patients with oligodendroglioma. Unfortunately, the number of patients with available, sequential [^18^F]FET PET imaging was low in this cohort. However, despite the low number of patients, there was a trend toward lower TBRmax values after treatment. As PET-derived markers have been shown to be associated with outcome in patients with IDH-mutant gliomas, TBRs and PET volume might aid in assessing residual tumor burden after therapy and, in case of neoadjuvant strategies, to define target volumes for resection and radiotherapy.^32^

Many patients included in this study were treated before the era of molecular classification and current treatment guidelines. At our institution, conservative treatment strategies had been frequently pursued, aiming to defer radiotherapy and the potentially associated long-term side effects. While many patients would be treated differently nowadays, the introduction of new tumor-specific treatments, such as IDH inhibitors, eg vorasidenib, improved risk stratification, and the oftentimes excellent survival times in patients with oligodendrogliomas might warrant room for individualized strategies in selected patients that could comprise deferral of radiotherapy or neoadjuvant PC. In this context, this study addresses the knowledge gap of radiological evolution and volume decrease under PC. Our study is limited by its retrospective single-center design, the modest cohort size, and the limited number of progression and death events. Accordingly, survival analyses, particularly multivariable Cox regression models, should be interpreted with caution, as effect estimates are subject to uncertainty and some confidence intervals were wide. Our findings should therefore be considered hypothesis-generating and require validation in larger independent cohorts. Furthermore, the high number of patients treated with temozolomide before PC in this cohort does not reflect the current standard of care for oligodendroglioma, but many patients included in the study had been diagnosed prior to the molecular classification in 2016 and prior to pivotal clinical studies.^24^^,^^33-35^ The preferred use of initial temozolomide at our department might stem from the favorable safety profile while still showing largely comparable efficacy to radiotherapy and PCV in the NOA-4 trial.^23^^,^^36^ However, according to newer studies, temozolomide might be associated with induction of tumor mutational burden and mismatch repair deficiency in some gliomas, and our data suggests inferior response in patients with prior chemotherapy.^15^^,^^37-40^ Further studies are needed to elucidate the efficacy and long-term effects of temozolomide in patients with oligodendroglioma. Our study suggests that prior chemotherapy and radiotherapy might impede the response to PC but does not answer the question whether there are differential effects of temozolomide or radiotherapy on response to PC. The patients included in this study were treated before introduction of vorasidenib, which showed promising results in patients with IDH-mutant, CNS WHO grade 2 gliomas.^8^ The response to PC after failure of vorasidenib therapy remains unknown.

In summary, PC polychemotherapy might be especially effective in reducing tumor volume in patients without prior radio- or temozolomide, and this effect does not appear to depend on absolute initial tumor size. PC might qualify as a neoadjuvant strategy in select patients to enable more extensive or even supramarginal tumor resections and to potentially allow reduction of radiation dose and field size to healthy brain parenchyma. Sharply delineated oligodendrogliomas might show earlier progression, and PC might shift the tumors toward a slightly more diffuse phenotype on MRI. The implication of diffusely versus sharply delineated phenotypes on MRI remains unclear, and correlations with histopathological findings are urgently needed.

## Supplementary Material

vdag184_Supplementary_Data

## Data Availability

The data analyzed in this study are not publicly available due to confidentiality and data protection regulations. Data may be made available on reasonable request and after approval by the local ethics committee.

## References

[1] Weller M , van den Bent M, Preusser M, et al EANO guidelines on the diagnosis and treatment of diffuse gliomas of adulthood. Nat Rev Clin Oncol. 2021;18:170-186. 10.1038/s41571-020-00447-z 33293629 PMC7904519

[2] Lassman AB , Iwamoto FM, Cloughesy TF, et al International retrospective study of over 1000 adults with anaplastic oligodendroglial tumors. Neuro Oncol. 2011;13:649-659. 10.1093/neuonc/nor040 21636710 PMC3107101

[3] Louis DN , Perry A, Wesseling P, et al The 2021 WHO classification of tumors of the central nervous system: a summary. Neuro Oncol. 2021;23:1231-1251. 10.1093/neuonc/noab106 34185076 PMC8328013

[4] Lebrun C , Fontaine D, Ramaioli A, et al; Nice Brain Tumor Study Group. Long-term outcome of oligodendrogliomas. Neurology. 2004;62:1783-1787. 10.1212/01.wnl.0000125196.88449.89 15159478

[5] Katzendobler S , Niedermeyer S, Blobner J, et al Determinants of long-term survival in patients with IDH-mutant gliomas. J Neurooncol. 2024;170:655-664. 10.1007/s11060-024-04826-9 39316316 PMC11614945

[6] Carstam L , Latini F, Solheim O, et al Long-term follow up of patients with WHO grade 2 oligodendroglioma. J Neurooncol. 2023;164:65-74. 10.1007/s11060-023-04368-6 37603235 PMC10462563

[7] Pignatti F , van den Bent M, Curran D, et al; European Organization for Research and Treatment of Cancer Radiotherapy Cooperative Group. Prognostic factors for survival in adult patients with cerebral low-grade glioma. J Clin Oncol. 2002;20:2076-2084. 10.1200/JCO.2002.08.121 11956268

[8] Mellinghoff IK , van den Bent MJ, Blumenthal DT, et al; INDIGO Trial Investigators. Vorasidenib in IDH1- or IDH2-mutant low-grade glioma. N Engl J Med. 2023;389:589-601. 10.1056/NEJMoa2304194 37272516 PMC11445763

[9] Leon-Ferre RA , Hieken TJ, Boughey JC. The landmark series: neoadjuvant chemotherapy for triple-negative and HER2-positive breast cancer. Ann Surg Oncol. 2021;28:2111-2119. 10.1245/s10434-020-09480-9 33486641 PMC8818316

[10] Conroy T , Castan F, Etienne P-L, et al Total neoadjuvant therapy with mFOLFIRINOX versus preoperative chemoradiotherapy in patients with locally advanced rectal cancer: long-term results of the UNICANCER-PRODIGE 23 trial. Ann Oncol. 2024;35:873-881. 10.1016/j.annonc.2024.06.019 38986769

[11] Pasquali S , Gronchi A. Neoadjuvant chemotherapy in soft tissue sarcomas: latest evidence and clinical implications. Ther Adv Med Oncol. 2017;9:415-429. 10.1177/1758834017705588 28607580 PMC5455882

[12] Forde PM , Spicer J, Lu S, et al; CheckMate 816 Investigators. Neoadjuvant nivolumab plus chemotherapy in resectable lung cancer. N Engl J Med. 2022;386:1973-1985. 10.1056/NEJMoa2202170 35403841 PMC9844511

[13] Bursi M , Rizzo C, Barberis M, et al Oncological, cognitive, and employment outcomes in a series of patients with IDH-mutated glioma resected following neoadjuvant chemotherapy. Acta Neurochir (Wien). 2023;165:2461-2471. 10.1007/s00701-023-05711-6 37482554

[14] Streffer J , Schabet M, Bamberg M, et al A role for preirradiation PCV chemotherapy for oligodendroglial brain tumors. J Neurol. 2000;247:297-302. 10.1007/s004150050587 10836623

[15] Weller J , Katzendobler S, Karschnia P, et al PCV chemotherapy alone for WHO grade 2 oligodendroglioma: prolonged disease control with low risk of malignant progression. J Neurooncol. 2021;153:283-291. 10.1007/s11060-021-03765-z 33932195 PMC8211617

[16] Diabira S , Rousselet MC, Gamelin E, Soulier P, Jadaud E, Menei P. PCV chemotherapy for oligodendroglioma: response analyzed on T2 weighted-MRI. J Neurooncol. 2001;55:45-50. 10.1023/a:1012986422074 11804282

[17] Webre C , Shonka N, Smith L, Liu D, De Groot J. PC or PCV, that is the question: primary anaplastic oligodendroglial tumors treated with procarbazine and CCNU with and without vincristine. Anticancer Res. 2015;35:5467-5472. 26408710

[18] Weller J , de Dios E, Katzendobler S, et al The T1/T2 ratio is associated with resectability in patients with isocitrate dehydrogenase-mutant astrocytomas central nervous system world health organization grades 2 and 3. Neurosurgery. 2025;96:365-372. 10.1227/neu.0000000000003069 38920377

[19] Ius T , Isola M, Budai R, et al Low-grade glioma surgery in eloquent areas: volumetric analysis of extent of resection and its impact on overall survival. A single-institution experience in 190 patients: clinical article. J Neurosurg. 2012;117:1039-1052. 10.3171/2012.8.JNS12393 23039150

[20] Skrap M , Mondani M, Tomasino B, et al Surgery of insular nonenhancing gliomas: volumetric analysis of tumoral resection, clinical outcome, and survival in a consecutive series of 66 cases. Neurosurgery. 2012;70:1081-1093. 10.1227/NEU.0b013e31823f5be5 22067417

[21] Weller J , Harba D, Kuschel L, et al Association of a diffuse phenotype on MRI with shorter overall survival in patients with astrocytoma CNS WHO grades 2 and 3. J Neurosurg. 2026;144:1419-1426. 10.3171/2025.9.JNS251254 41616295

[22] Nigro JM , Takahashi MA, Ginzinger DG, et al Detection of 1p and 19q loss in oligodendroglioma by quantitative microsatellite analysis, a real-time quantitative polymerase chain reaction assay. Am J Pathol. 2001;158:1253-1262. 10.1016/S0002-9440(10)64076-X 11290543 PMC1891922

[23] Wick W , Hartmann C, Engel C, et al NOA-04 randomized phase III trial of sequential radiochemotherapy of anaplastic glioma with procarbazine, lomustine, and vincristine or temozolomide. J Clin Oncol. 2009;27:5874-5880. 10.1200/JCO.2009.23.6497 19901110

[24] van den Bent MJ , Brandes AA, Taphoorn MJB, et al Adjuvant procarbazine, lomustine, and vincristine chemotherapy in newly diagnosed anaplastic oligodendroglioma: long-term follow-up of EORTC brain tumor group study 26951. J Clin Oncol. 2013;31:344-350. 10.1200/JCO.2012.43.2229 23071237

[25] Katzendobler S , Do A, Weller J, et al Diagnostic yield and complication rate of stereotactic biopsies in precision medicine of gliomas. Front Neurol. 2022;13:822362. 10.3389/fneur.2022.822362 35432168 PMC9005817

[26] Katzendobler S , Do A, Weller J, et al The value of stereotactic biopsy of primary and recurrent brain metastases in the era of precision medicine. Front Oncol. 2022;12:1014711. 10.3389/fonc.2022.1014711 36605448 PMC9808072

[27] Wen PY , van den Bent M, Youssef G, et al RANO 2.0: update to the response assessment in Neuro-Oncology criteria for high- and low-grade gliomas in adults. J Clin Oncol. 2023;41:5187-5199. 10.1200/JCO.23.01059 37774317 PMC10860967

[28] US Department of Health and Human Services; National Institutes of Health NCI. Common Terminology Criteria for Adverse Events (CTCAE) Version 5. 2017.

[29] Albert NL , Galldiks N, Ellingson BM, et al PET-based response assessment criteria for diffuse gliomas (PET RANO 1.0): a report of the RANO group. Lancet Oncol. 2024;25:e29-e41. 10.1016/S1470-2045(23)00525-9 38181810 PMC11787868

[30] Hervey-Jumper SL , Zhang Y, Phillips JJ, et al Interactive effects of molecular, therapeutic, and patient factors on outcome of diffuse low-grade glioma. J Clin Oncol. 2023;41:2029-2042. 10.1200/JCO.21.02929 36599113 PMC10082290

[31] Karschnia P , Young JS, Wijnenga MMJ, et al A prognostic classification system for extent of resection in IDH-mutant grade 2 glioma: an international, multicentre, retrospective cohort study with external validation by the RANO resect group. Lancet Oncol. 2025;26:1638-1650. 10.1016/S1470-2045(25)00534-0 41308678

[32] Mair MJ , Werner J-M, Weller J, et al Prognostic stratification of newly diagnosed IDH-mutant gliomas by [18F]fluoroethyltyrosine and [11C]methionine PET - a retrospective, bicentric cohort study. Neuro Oncol. 2025;27:3292-3305. 10.1093/neuonc/noaf196 40856188 PMC12916739

[33] Buckner JC , Chakravarti A, Curran WJ. Jr., Radiation plus chemotherapy in low-grade glioma. N Engl J Med. 2016;375:490-491. 10.1056/NEJMc1605897 27518677

[34] Cairncross G , Wang M, Shaw E, et al Phase III trial of chemoradiotherapy for anaplastic oligodendroglioma: long-term results of RTOG 9402. J Clin Oncol. 2013;31:337-343. 10.1200/JCO.2012.43.2674 23071247 PMC3732012

[35] Bell EH , Zhang P, Shaw EG, et al Comprehensive genomic analysis in NRG oncology/RTOG 9802: a phase III trial of radiation versus radiation plus procarbazine, lomustine (CCNU), and vincristine in high-risk low-grade glioma. J Clin Oncol. 2020;38:3407-3417. 10.1200/JCO.19.02983 32706640 PMC7527157

[36] Wick W , Roth P, Hartmann C, et al; Neurooncology Working Group (NOA) of the German Cancer Society. Long-term analysis of the NOA-04 randomized phase III trial of sequential radiochemotherapy of anaplastic glioma with PCV or temozolomide. Neuro Oncol. 2016;18:1529-1537. 10.1093/neuonc/now133 27370396 PMC5063521

[37] Touat M , Li YY, Boynton AN, et al Mechanisms and therapeutic implications of hypermutation in gliomas. Nature. 2020;580:517-523. 10.1038/s41586-020-2209-9 32322066 PMC8235024

[38] Barthel FP , Johnson KC, Varn FS, et al; GLASS Consortium. Longitudinal molecular trajectories of diffuse glioma in adults. Nature. 2019;576:112-120. 10.1038/s41586-019-1775-1 31748746 PMC6897368

[39] van Thuijl HF , Mazor T, Johnson BE, et al Evolution of DNA repair defects during malignant progression of low-grade gliomas after temozolomide treatment. Acta Neuropathol. 2015;129:597-607. 10.1007/s00401-015-1403-6 25724300 PMC4482618

[40] Yu Y , Villanueva-Meyer J, Grimmer MR, et al Temozolomide-induced hypermutation is associated with distant recurrence and reduced ­survival after high-grade transformation of low-grade IDH-mutant ­‑gliomas. Neuro Oncol. 2021;23:1872-1884. 10.1093/neuonc/noab081 33823014 PMC8563321

