# Supplementary material for: Patterns of radiological response to procarbazine and CCNU polychemotherapy in patients with oligodendroglioma
Source: Neurooncol Adv. 2026 Aug 6;8(1):vdag184. doi: 10.1093/noajnl/vdag184 (PMC13476079; doi:10.1093/noajnl/vdag184)
Supplement: Supplementary file 1 [file vdag184_supplementary_data.docx]

# **Supplementary material**

Suppl. Fig. 1. **Patient selection.** Patients diagnosed between 2003 and 2019 were included. *IDH, isocitrate dehydrogenase gene 1 or 2; CNS, central nervous system; WHO, World Health Organization; PC, procarbazine and CCNU (lomustine).*

Suppl. table 1: Baseline characteristics stratified by response (RANO 2.0)

| **Parameter** | **Partial or minor response**  **(n=11)** | **Stable Disease**  **(n=29)** | **Progressive Disease**  **(n=6)** | ***p*-value** |
| --- | --- | --- | --- | --- |
| **Age (years)** |  |  |  |  |
| Median | 33 | 39 | 41 | *0.79* |
| Range | 19-64 | 19-75 | 26-48 |  |
| **Sex, n (%)** |  |  |  |  |
| Female | 7 (64) | 10 (35) | 3 (50) | *0.24* |
| Male | 4 (36) | 19 (66) | 3 (50) |  |
| **CNS WHO grade, n (%)** |  |  |  |  |
| 2 | 10 (91) | 24 (83) | 5 (83) | *0.89* |
| 3 | 1 (9) | 5 (17) | 1 (17) |  |
| **Contrast enhancement, n (%)** |  |  |  |  |
| No | 6 (55) | 9 (31) | 0 (0) | *0.19* |
| Mild/minimal | 2 (18) | 11 (38) | 4 (66) |  |
| Marked/avid | 3 (27) | 9 (31) | 2 (33) |  |
| **Initial T2 volume (cm^3^)** |  |  |  |  |
| Median | 37 | 69 | 52 | *0.13* |
| Range | 10-116 | 14-285 | 10-134 |  |
| **Prior tumor-specific therapies, n (%)** |  |  |  |  |
| 0 | 6 (55) | 7 (24) | 0 (0) | *0.02** |
| 1 | 3 (27) | 11 (38) | 0 (0) |  |
| 2 | 2 (18) | 5 (17) | 3 (50) |  |
| ≥3 | 0 (0) | 6 (21) | 3 (50) |  |
| **Initial T1/T2 ratio** |  |  |  |  |
| Median | 0.71 | 0.61 | 0.81 | *0.67* |
| Range | 0.16-1.0 | 0.06-1.0 | 0.03-1.0 |  |

Prior tumor-specific therapies comprise tumor resection, radiotherapy and temozolomide chemotherapy. *RANO, Response Assessment in Neuro-Oncology; CNS, central nervous system; WHO, World Health Organization.*

Supplementary table 2: Uni- and multivariate analyses for post-PC survival

| **Univariate analyses** |  |  |  |
| --- | --- | --- | --- |
| **Factor** | **Post-PC survival** |  |  |
|  | **HR** | **95% CI** | **p-value** |
| **Age^x^** | 1.01 | 0.96-1.07 | *0.59* |
| **KPS^x^** | 0.91 | 0.86-0.97 | ***<0.01**** |
| **CNS WHO grade (3 versus 2)** | 0.95 | 0.26-6.07 | *0.95* |
| **Contrast enhancement (marked versus minimal versus no)** | 0.42 | 0.2-0.83 | ***0.01**** |
| **T2 volume before PC^x^** | 1.00 | 1.0-1.01 | *0.22* |
| **T2 volume after PC^x^** | 1.01 | 1.0-1.02 | ***0.02**** |
| **Volume change (absolute) ^x^** | 1.01 | 1.0-1.02 | *0.28* |
| **Volume change (relative) ^x^** | 2.14 | 0.74-5.57 | *0.15* |
| **T1/T2 ratio before PC^x^** | 0.76 | 0.17-3.48 | *0.72* |
| **T1/T2 ratio after PC^x^** | 4.45 | 0.94-23.99 | *0.06* |
| **PC cycles^(n)^** | 0.77 | 0.56-1.11 | *0.16* |
| **Prior tumor-specific therapies^x^** | 1.57 | 1.18-2.05 | ***<0.01**** |
| **Multivariate analyses** |  |  |  |
| **Factor** | **Post-PC survival** |  |  |
|  | **HR** | **95% CI** | **p-value** |
| **KPS^x^** | 0.97 | 0.9-1.06 | *0.47* |
| **Contrast enhancement (marked versus minimal versus no)** | 0.5 | 0.18-1.34 | *0.17* |
| **T2 volume after PC^x^** | 1.01 | 1.0-1.02 | ***0.03**** |
| **Prior tumor-specific therapies^x^** | 1.35 | 0.88-2.04 | *0.16* |

Prior tumor-specific therapies comprise tumor resection, radiotherapy and temozolomide chemotherapy. *PC, procarbazine and CCNU (lomustine); OS, overall survival; HR, hazard ratio; CI, confidence interval; KPS, Karnofsky performance status; CNS, central nervous system; WHO, World Health Organization; n, number of cycles. Statistical significance: p < 0.05 (*). Continuously scaled variables are denoted by* **^x^.**
